# Perception of Western Musical Modes: A Chinese Study

**DOI:** 10.3389/fpsyg.2017.01905

**Published:** 2017-10-31

**Authors:** Lele Fang, Junchen Shang, Nan Chen

**Affiliations:** ^1^School of Psychology, Liaoning Normal University, Dalian, China; ^2^Cooperative Innovation Center of Healthy Personality Evaluation and Cultivation of Children and Adolescents in Liaoning Province, Dalian, China

**Keywords:** mode, harmonic complexity, culture, perception, emotion

## Abstract

The major mode conveys positive emotion, whereas the minor mode conveys negative emotion. However, previous studies have primarily focused on the emotions induced by Western music in Western participants. The influence of the musical mode (major or minor) on Chinese individuals’ perception of Western music is unclear. In the present experiments, we investigated the effects of musical mode and harmonic complexity on psychological perception among Chinese participants. In Experiment 1, the participants (*N* = 30) evaluated 24 musical excerpts in five dimensions (pleasure, arousal, dominance, emotional tension, and liking). In Experiment 2, the participants (*N* = 40) evaluated 48 musical excerpts. Perceptions of the musical excerpts differed significantly according to mode, even if the stimuli were Western musical excerpts. The major-mode music induced greater pleasure and arousal and produced higher liking ratings than the minor-mode music, whereas the minor-mode music induced greater tension than the major-mode music. Mode did not influence the dominance rating. Perception of Western music was not influenced by harmonic complexity. Moreover, preference for musical mode was influenced by previous exposure to Western music. These results confirm the cross-cultural emotion induction effects of musical modes in Western music.

## Introduction

Music is one of the most relevant forms of human artistic expression, as it is relatable across a wide range of different life situations. This characteristic has made music a topic of interest in the field of psychology. Brattico and Pearce (2013) suggested that the evaluation of music should be based on an individual’s musical perceptions, including music-induced emotions, judgments, and preferences. Recently, many researchers have explored musical perceptions (Juslin and Västfjäll, 2008; Brattico and Jacobsen, 2009; Brattico et al., 2010, 2013; Juslin, 2013; Juslin and Isaksson, 2014; Koelsch, 2014). The structural features of music and the listeners’ features can influence music-evoked emotions (Juslin and Lindström, 2010; Garrido and Schubert, 2011; Vuoskoski and Eerola, 2011; Ladinig and Schellenberg, 2012; Vuoskoski et al., 2012; Silvia et al., 2015). However, previous studies have primarily involved Westerner listeners and musical stimuli belonging to the Western musical tradition. Whether the factors that influence music-induced emotions in Westerner listeners also play a role in the musical perceptions of Chinese people remains unknown. The current studies explored the effects of the music mode and the complexity of the musical semantics on music-induced emotions and liking ratings among Chinese people.

The structural features of music influence psychological perceptions of Western music (Juslin and Lindström, 2010). The mode is one of the most important structural features that constitute the expressional characteristics of music. The melody is the linear succession of musical tones that vary in pitch. The mode is a structural feature embodied in the structural relationship among the tones that constitute the basic series of music. The major and minor modes are widely used in music. The structural difference between the major mode and the minor mode produces distinct sound effects. The root of the minor triad is more ambiguous and uncertain than the root of the major triad (Parncutt, 2014); therefore, the major mode tends to induce positive emotions, while the minor mode tends to induce negative emotions (Juslin and Lindström, 2010; Huron and Davis, 2012; Juslin and Sloboda, 2013; Straehley and Loebach, 2014). For example, Lahdelma and Eerola (2014) explored the emotional perceptions of different chords (i.e., major, minor, diminished, augmented, and seventh chords) and showed that the valence ratings of the major triad were the highest. Some studies have also investigated individual psychological differences in the perception of musical mode. Bonetti and Costa (2017) tracked the development of cross-modal associations between musical mode and emotional-visual-spatial dimensions in children and adults. They found that 6-year-old children and adults rated major-mode music as more pleasant than minor-mode music. In addition, the preference for minor-mode music was associated with fluid intelligence and openness to experiences (Bonetti and Costa, 2016).

Although several studies have explored the influence of music on emotion, the methods used to categorize and measure emotions differed. Therefore, comparing the findings of these studies is difficult. More studies are needed to reach a solid conclusion. Some studies categorized the basic emotions induced by music, such as happiness, fear, sadness, anger, etc. (Balkwill and Thompson, 1999; Balkwill et al., 2004; Fritz et al., 2009; Zacharopoulou and Kyriakidou, 2009; Trost et al., 2012). However, this categorization is too simple and does not fully capture the complexity of the emotions expressed by music. Moreover, the categorization of emotions into several basic emotions is controversial (Barrett, 2006; Lindquist et al., 2013). In contrast to the categorical framework of basic emotions, the multi-dimensional emotion model is more detailed and covers all basic emotions (Brattico and Pearce, 2013). For instance, several studies have used the two-dimensional emotion model of arousal and valence to examine the emotion induction effects of music (Brattico and Pearce, 2013; Droit-Volet et al., 2013; Egermann et al., 2015; Grekow, 2016). Other studies have used the three-dimensional emotional model of valence, arousal, and tension (Schimmack and Grob, 2000; van der Zwaag et al., 2011; Brattico and Pearce, 2013). Dominance is another important dimension in emotion that represents a person’s control over a situation and other people. The stronger the feeling of control, the higher the dominance rating (Mehrabian, 1995, 1996; Mehrabian et al., 1997). The three dimensions of pleasure, arousal, and dominance constitute the PAD emotional model, which has been broadly applied to certain research areas, such as emotion, facial perception, and product satisfaction (Bradley and Lang, 1994; Backs et al., 2005; Zhang et al., 2011; Shang et al., 2013). However, to date, few studies have explored the effect of music on dominance.

Moreover, most studies investigating the musical perception of mode have focused on Western music and Western people. It is unclear whether a relationship exists between mode and music perception is unknown among Chinese people, particularly for music belonging to the Western musical tradition. Currently, cultural differences in musical perception are controversial. Commonalities in music-induced emotions among listeners from different cultures have been reported. Balkwill and Thompson (1999) reported that Western listeners were sensitive to the emotion conveyed by Hindustani music. Moreover, Balkwill et al. (2004) have shown that Japanese participants were sensitive to the emotions conveyed by Japanese, Western and Hindustani music. Balkwill et al. (2004) suggested that acoustic cues can be cross-culturally understood and influence the emotion induced by music. Fritz et al. (2009) have also found that Mafa listeners who were naïve to Western music recognized happy, sad, and fearful emotions in Western music at a level greater than chance. In contrast to the findings reported by Fritz et al. (2009), Egermann et al. (2015) reported that the arousal ratings were similar between Canadians and Pygmies who were naïve to Western music, whereas the valence ratings of the music differed between the two groups. However, few studies examined the influence of the mode on the perception of music from unfamiliar cultures, except for Hoshino (1996). Hoshino reported that the major mode produces different emotional reactions than the minor mode in Japanese listeners. Furthermore, the above-mentioned studies examined only the emotions induced by music; whether there is a difference in the liking ratings of music with different modes from unfamiliar cultures is unclear. In addition, these studies all used different experimental paradigms, materials and measurements; thus, reaching a conclusion is difficult.

In addition to the musical modes, the complexity of the musical semantics influences emotion induction. Bigand et al. (1996) reported that the chord sequence “tonic–tonic–tonic” (t–t–t) induced less tension than the sequence “tonic–dominant–tonic” (t–d–t). Thus, music with slightly higher harmonic variance and complexity produced greater tension. However, tension is not sufficient for describing the emotion induction effect in terms of the variety of emotion. The current study aimed to examine the influence of the mode and harmonic complexity of Western music on the psychological perception of Chinese people. The participants evaluated music according to the following five dimensions: pleasure, arousal, dominance, tension of the emotion and liking ratings. If the mode and harmonic variance of the Western music had cross-cultural effects on music perception in the Chinese participants, music in the major mode was hypothesized to induce more positive emotions than the music in the minor mode. The differences in the harmonic complexity were also hypothesized to result in differences in the emotion induction effects. Moreover, the differences in the mode and harmonic complexity were hypothesized to lead to differences in the liking ratings.

## Experiment 1

### Materials and Methods

#### Participants

Thirty Chinese university students (15 male, *M*_age_ = 20.76, *SD*_age_ = 1.45) volunteered to participate in the experiment. All participants were raised in China. They had normal hearing and no cognitive disabilities. The sociodemographic data of the participants, including age, annual household income, years of education, and years of musical training, are reported in **Table 1**. Six participants had more than 2 years of specific musical training and were excluded from further analysis. The remaining participants (*N* = 24) did not have any specific musical training. We asked the remaining participants about their familiarity with Western music. Ten participants reported a minor amount of exposure to Western music as interested listeners (on average 6.30 h/week) but no knowledge of the Western musical tonal system. Nine participants occasionally listened to Western music (on average 1.44 h/week) but had no knowledge of the Western musical tonal system. Three participants indicated no familiarity with Western music. The descriptive data about music listening were missing for two participants. Therefore, the main analysis included 22 participants. All subjects provided written informed consent. The study protocol was approved by the Institutional Review Board of Liaoning Normal University.

**Table 1:** Demographic characteristics of the participants in Experiments 1 and 2.

	Gender	Age	Education (years)	Musical learning (years)	Annual household income (US dollars)
Experiment 1	Male	21.06 (1.82)	13.88 (2.09)	1.69 (3.18)	12835 (6942)
	Female	20.43 (0.62)	13.82 (0.49)	0.57 (1.59)	11604 (4988)
Experiment 2	Male	22.60 (2.29)	15.83 (1.73)	0	12421 (6750)
	Female	21.90 (1.97)	15.47 (1.33)	0	12436 (7478)

*The values in the table are the means, and the standard deviations are presented in the parentheses.*

#### Stimuli and Procedures

The stimuli were displayed on a Lenovo computer with a 17-inch LCD monitor at a 60 Hz refresh rate and a screen resolution of 1440 × 900 pixels. The E-prime 2.0 software package was used for the stimuli presentation and data collection.

**FIGURE 1:**
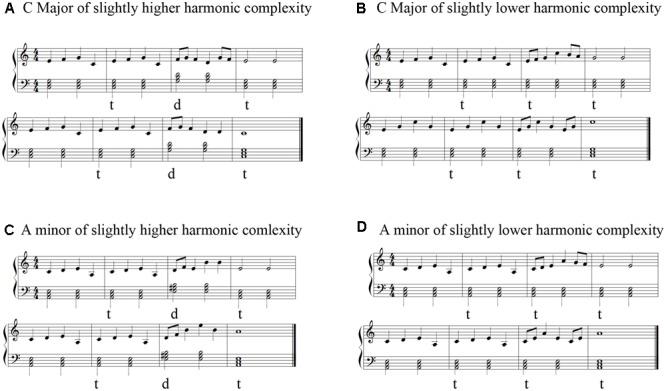
Examples of the experimental stimuli. There were four versions of each piano sonata as follows: **(A)** “C major”/slightly higher harmonic complexity (t–d–t); **(B)** “C major”/slightly lower harmonic complexity (t–t–t); **(C)** “A minor”/slightly higher harmonic complexity (t–d–t); and **(D)** “A minor”/slightly lower harmonic complexity (t–t–t).

A graduate student majoring in musical composition was asked to select six musical excerpts from the “Bayer Piano Basic Course.” Each musical excerpt had eight bars. First, each excerpt was modified into two versions. One version was in the major mode, and the other version was in the minor mode. The “C major” and “A minor” modes were selected. Second, two of the eight bars in each excerpt were modified into the following two harmonic sequences: t–t–t and t–d–t. The insertion of the dominant chords resulted in a slightly higher level of harmonic-semantic variance than that observed in the condition with only the tonic chords. The t–t–t sequence is less complex than the t–d–t sequence. Thus, each original excerpt was modified into the following four versions: “C major”/higher harmonic complexity (t–d–t), “C major”/lower harmonic complexity (t–t–t), “A minor”/higher harmonic complexity (t–d–t) and “A minor”/lower harmonic complexity (t–t–t). The minor stimuli were always ascribable to the “harmonic scale.” In total, there were 24 excerpts. To avoid any differences in the sound effects due to the inversion chord (Lahdelma and Eerola, 2014), the current study only adopted the triad chord in the root position. The excerpts were generated using Encore software (which is used to generate piano sonatas) as MIDI files. All modified excerpts were in 4/4 meters. The sound levels were between 65 and 75 decibels (dB). The tempo of each excerpt was 88 beats per minute (bpm). Each excerpt had a duration of 22 s. Examples of the musical stimuli are shown in **Figure 1**. To avoid any changes in the fluency of the music, two graduate students majoring in musical performance were asked to rate the fluency of the modified music using a five-point scale (1–5). The ratings of the musical materials were all above 2.5. Therefore, the fluency of the music stimuli was good.

The Self-Assessment Manikin (SAM, Bradley and Lang, 1994; Morris, 1995) was used to measure the pleasure, arousal, and dominance of the emotion induced by the music. Each dimension was measured on a continuous nine-point scale (ranging from 1 to 9, low to high). The SAM is a picture scale that can be directly used to measure the three basic dimensions of emotion (Bradley and Lang, 1994). For pleasure, the SAM ranges from a frowning, unhappy figure to a smiling, happy figure. For arousal, the SAM ranges from a relaxed, sleepy figure to an excited, wide-eyed figure. For dominance, the SAM ranges from a small figure (i.e., minimal situational control) to a large figure (maximum situational control). Moreover, the rating of tension and liking rating relied on nine-point scales (ranging from 1 to 9, low to high). According to previous studies, tension was interpreted as a feeling of instability, disharmony, and surprise (Krumhansl, 1996; Lerdahl and Krumhansl, 2007; Koelsch et al., 2008). For the liking rating, the participants were asked to answer the following question: “How much did you like the music?”

First, the participants entered the lab and completed a demographic questionnaire. Second, the experimenter helped the participants wear the headphones (Sennheiser HD201) and adjust their sitting postures. Two practice trials were performed before the formal experiment. Twenty-four excerpts were played in a random order. After each excerpt, the participants were asked to evaluate the dimensions of pleasure, arousal, dominance, and tension of the emotion induced by the music and complete their liking ratings. After the experiment, the participants were debriefed and thanked for their participation.

### Statistical Analysis

The experimental design was a 2 (modes: major vs. minor) × 2 (harmonic complexity: higher vs. lower) within-subjects design. The independent variables were the mode and harmonic complexity. The dependent variables were the scores of pleasure, arousal, dominance, and tension and the liking ratings. Analyses of covariance (ANCOVAs) were conducted, taking the number of hours of Western music listening as a covariate. We performed five three-way ANCOVAs for the number of hours of Western music listening, with modes (major vs. minor) and harmonic complexity (higher vs. lower) included as within-subject factors.

### Results

The ratings for the music differing in mode and harmonic complexity are shown in **Table 2**. Regarding pleasure, the main effect of mode was significant, *F*(1,83) = 24.51, *p* < 0.001, $\eta_{p}^{2}$ = 0.23, and the major-mode music induced more pleasure than the minor-mode music. The main effect of harmonic complexity was not significant, *F*(1,83) = 0.11, *p* = 0.739, $\eta_{p}^{2}$ = 0.001. The interaction between the mode and harmonic complexity was not significant, *F*(1,83) = 0.04, *p* = 0.841, $\eta_{p}^{2}$ = 0.0005. The covariate was not significant, *F*(1,83) = 3.31, *p* = 0.073, $\eta_{p}^{2}$ = 0.04.

**Table 2:** The mean ratings of the musical excerpts in Experiment 1.

Dimensions of psychological perception	Major mode		Minor mode	
	Higher complexity	Lower complexity	Higher complexity	Lower complexity
Pleasure	5.65 (0.96)	5.66 (0.89)	4.66 (1.22)	4.58 (1.25)
Arousal	5.00 (1.18)	5.10 (1.02)	4.61 (1.15)	4.51 (1.17)
Dominance	5.17 (0.91)	5.26 (0.96)	4.72 (0.85)	4.74 (1.12)
Tension	3.15 (0.84)	3.58 (1.03)	4.38 (1.35)	4.54 (1.39)
Liking ratings	5.88 (1.26)	5.30 (1.30)	5.42 (1.23)	4.74 (1.54)

*The values in the table are the mean ratings of the music under each condition. The standard deviations are presented in the parentheses. Scale values ranged from 1 (*low on a dimension*) to 9 (*high on a dimension*).*

Regarding arousal, the main effect of mode was significant, *F*(1,83) = 4.30, *p* = 0.041, $\eta_{p}^{2}$ = 0.05, and the major-mode music induced greater arousal than the minor-mode music. The main effect of harmonic complexity was not significant, *F*(1,83) = 0.05, *p* = 0.832, $\eta_{p}^{2}$= 0.001. The interaction between the mode and harmonic complexity was not significant, *F*(1,83) = 0.18, *p* = 0.671, $\eta_{p}^{2}$ = 0.002. The covariate was not significant, *F*(1,83) = 0.99, *p* = 0.322, $\eta_{p}^{2}$ = 0.01.

Regarding dominance, the main effect of mode was significant, *F*(1,83) = 5.91, *p* = 0.017, $\eta_{p}^{2}$ = 0.07, and the major-mode music induced greater dominance than the minor-mode music. The main effect of harmonic complexity was not significant, *F*(1,83) = 0.01, *p* = 0.914, $\eta_{p}^{2}$ = 0.0001. The interaction between the mode and harmonic complexity was not significant, *F*(1,83) = 0.01, *p* = 0.942, $\eta_{p}^{2}$ = 0.00006. The covariate was not significant, *F*(1,83) = 0.03, *p* = 0.874, $\eta_{p}^{2}$ = 0.0003.

Regarding tension, the main effect of mode was significant, *F*(1,83) = 21.64, *p* < 0.001, $\eta_{p}^{2}$= 0.21, and the minor-mode music induced greater tension than the major-mode music. The main effect of harmonic complexity was not significant, *F*(1,83) = 1.52, *p* = 0.221, $\eta_{p}^{2}$= 0.02. The interaction between the mode and harmonic complexity was not significant, *F*(1,83) = 0.26, *p* = 0.612, $\eta_{p}^{2}$ = 0.003. The covariate was not significant, *F*(1,83) = 2.50, *p* = 0.118, $\eta_{p}^{2}$ = 0.03.

Regarding the liking ratings, the main effect of mode was not significant, *F*(1,83) = 3.30, *p* = 0.073, $\eta_{p}^{2}$ = 0.04. The main effect of harmonic complexity was significant, *F*(1,83) = 5.89, *p* = 0.017, $\eta_{p}^{2}$ = 0.07, and the music with a slightly higher complexity was more liked than the music with a slightly lower complexity. The interaction between the mode and harmonic complexity was not significant, *F*(1,83) = 0.0002, *p* = 0.989, $\eta_{p}^{2}$ = 0.000002. The covariate was significant, *F*(1,83) = 16.19, *p* < 0.001, $\eta_{p}^{2}$ = 0.16.

### Discussion

Experiment 1 showed that the mode and harmonic complexity influenced the psychological perception of Western music in Chinese people. Consistent with previous studies (Juslin and Lindström, 2010; Huron and Davis, 2012; Juslin and Sloboda, 2013; Parncutt, 2014; Straehley and Loebach, 2014; Lahdelma and Eerola, 2014; Bonetti and Costa, 2017), the major mode induced greater pleasure than the minor mode. Moreover, the major mode induced greater arousal and dominance than the minor mode, whereas the minor mode induced greater tension than the major mode. In addition, slightly more complex music induced greater liking ratings than less complex music. The number of hours of Western music listening also influenced liking ratings, but it did not influence other perceptions.

Experiment 1 was limited because the number of stimuli and the sample size were small. Therefore, to increase the reliability of our results, we decided to replicate our findings by performing a further experiment with an increased number of musical excerpts. Specifically, we recruited 40 participants in Experiment 2. Each condition included 12 excerpts.

## Experiment 2

### Materials and Methods

#### Participants

Forty Chinese university students (10 male, *M*_age_ = 22.08, *SD*_age_ = 2.07) volunteered to participate in the experiment. All participants had normal hearing and no cognitive disabilities. The participants did not have any specific musical training. The sociodemographic data of the participants, including age, annual household income, years of education, and years of music training, are reported in **Table 1**. We asked the participants about their familiarity with Western music. Four of them reported a minor amount of exposure to Western music as interested listeners (on average 8.50 h/week) but no knowledge of the Western musical tonal system. Twenty-eight participants occasionally listened to Western music (on average 1.61 h/week) but had no knowledge of the Western musical tonal system. Seven participants indicated no familiarity with Western music. The descriptive data about music listening were missing for one participant. Thus, the main analysis included 39 participants. All subjects provided written informed consent. The study protocol was approved by the Institutional Review Board of Liaoning Normal University.

#### Stimuli and Procedures

The stimuli and procedures were identical to those in Experiment 1, except for the inclusion of 12 excerpts in each condition (48 excerpts in total).

### Statistical Analysis

The experimental design was a 2 (modes: major vs. minor) × 2 (harmonic complexity: higher vs. lower) within-subjects design. The independent variables were the mode and harmonic complexity. The dependent variables were the pleasure, arousal, dominance, tension, and liking ratings. ANCOVAs were conducted by taking the number of hours of Western music listening as a covariate. We performed five three-way ANCOVAs for the number of hours of Western music listening, with modes (major vs. minor) and harmonic complexity (higher vs. lower) included as within-subjects factors.

### Results

The ratings of the music, which differed between the mode and harmonic complexity, are shown in **Table 3**.

**Table 3:** The mean ratings of the musical excerpts in Experiment 2.

Dimensions of psychological perception	Major mode		Minor Mode	
	Higher complexity	Lower complexity	Higher complexity	Lower complexity
Pleasure	5.77 (1.06)	5.59 (1.03)	4.98 (0.99)	4.94 (1.01)
Arousal	5.24 (0.89)	5.15 (0.99)	4.80 (0.97)	4.76 (0.88)
Dominance	4.86 (1.03)	4.69 (1.00)	4.58 (0.97)	4.45 (0.94)
Tension	3.69 (1.10)	3.89 (1.12)	4.34 (1.29)	4.38 (1.28)
Liking ratings	5.52 (1.31)	5.22 (1.22)	4.93 (1.43)	4.78 (1.35)

*The values in the table are the mean ratings of the music under each condition. The standard deviations are presented in the parentheses. Scale values ranged from 1 (*low on a dimension*) to 9 (*high on a dimension*).*

Regarding pleasure, the main effect of mode was significant, *F*(1,151) = 21.21, *p* < 0.001, $\eta_{p}^{2}$ = 0.12, and the major mode induced greater pleasure than the minor mode. The main effect of harmonic complexity was not significant, *F*(1,151) = 0.55, *p* = 0.459, $\eta_{p}^{2}$ = 0.004. The interaction between the mode and harmonic complexity was not significant, *F*(1,151) = 0.22, *p* = 0.637, $\eta_{p}^{2}$ = 0.001. The covariate was significant, *F*(1,151) = 11.17, *p* = 0.001, $\eta_{p}^{2}$ = 0.07, suggesting that the number of hours of Western music listening influenced pleasure ratings.

Regarding arousal, the main effect of mode was significant, *F*(1,151) = 8.00, *p* = 0.005, $\eta_{p}^{2}$ = 0.05, and the major-mode music induced greater arousal than the minor-mode music. The main effect of harmonic complexity was not significant, *F*(1,151) = 0.24, *p* = 0.624, $\eta_{p}^{2}$= 0.002. The interaction between the mode and harmonic complexity was not significant, *F*(1,151) = 0.03, *p* = 0.863, $\eta_{p}^{2}$ = 0.0002. The covariate was not significant, *F*(1,151) = 0.06, *p* = 0.813, $\eta_{p}^{2}$ = 0.0004.

Regarding dominance, the main effect of mode was not significant, *F*(1,151) = 2.74, *p* = 0.10, $\eta_{p}^{2}$ = 0.02. The main effect of harmonic complexity was not significant, *F*(1,151) = 1.04, *p* = 0.309, $\eta_{p}^{2}$ = 0.007. The interaction between the mode and harmonic complexity was not significant, *F*(1,151) = 0.02, *p* = 0.887, $\eta_{p}^{2}$ = 0.0001. The covariate was not significant, *F*(1,151) = 0.01, *p* = 0.91, $\eta_{p}^{2}$ = 0.00008.

Regarding tension, the main effect of mode was significant, *F*(1,151) = 9.30, *p* = 0.003, $\eta_{p}^{2}$= 0.06, and the minor-mode music induced more tension than the major-mode music. The main effect of harmonic complexity was not significant, *F*(1,151) = 0.33, *p* = 0.568, $\eta_{p}^{2}$= 0.002. The interaction between the mode and harmonic complexity was not significant, *F*(1,151) = 0.14, *p* = 0.709, $\eta_{p}^{2}$ = 0.001. The covariate was not significant, *F*(1,151) = 1.07, *p* = 0.304, $\eta_{p}^{2}$ = 0.007.

Regarding the liking ratings, the main effect of mode was significant, *F*(1,151) = 6.19, *p* = 0.014, $\eta_{p}^{2}$ = 0.04, and the major-mode music was more liked than the minor-mode music. The main effect of harmonic complexity was not significant, *F*(1,151) = 1.14, *p* = 0.287, $\eta_{p}^{2}$ = 0.008. The interaction between the mode and harmonic complexity was not significant, *F*(1,151) = 0.06, *p* = 0.803, $\eta_{p}^{2}$ = 0.0004. The covariate was not significant, *F*(1,151) = 3.25, *p* = 0.074, $\eta_{p}^{2}$ = 0.02.

### Discussion

Experiment 2 replicated the primary findings observed in Experiment 1 except those for pleasure, dominance, and liking. The number of hours of Western music listening influenced pleasure ratings but did not influence liking ratings and other perceptions. The major mode induced greater pleasure and produced higher liking ratings than did the minor mode. However, in contrast to the results of Experiment 1, mode did not influence the dominance rating. In addition, harmonic complexity did not influence liking ratings. The different results may be due to the different number of stimuli and the size of the sample. The results observed in Experiment 2 were more reliable because the sample size and number of stimuli were larger than those in Experiment 1. We discuss the possible explanations for these findings in the following section.

## General Discussion

### The Influence of Musical Mode on Psychological Perceptions

The present study investigated the psychological perceptions of Western musical modes in Chinese participants. Two experiments were performed, and both showed a robust effect for mode. Consistent with studies in Western participants (Juslin and Lindström, 2010; Huron and Davis, 2012; Juslin and Sloboda, 2013; Parncutt, 2014; Straehley and Loebach, 2014; Lahdelma and Eerola, 2014; Bonetti and Costa, 2017), the major-mode music induced more pleasure than the minor-mode music. Moreover, the major-mode music received higher liking ratings. The participants preferred listening to major-mode music over minor-mode music, which may be because the music liking ratings were related to positive emotional experiences (Brattico and Pearce, 2013) induced by the major-mode music. Our findings are also consistent with those reported by Hoshino (1996), who reported that Japanese listeners associated the major mode with positive emotions and the minor mode with negative emotions. Regarding pleasure, the Western musical excerpts clearly induced similar perceptions in the Chinese sample, which was consistent with Western studies involving Western samples. This study is consistent with the studies by Balkwill and Thompson (1999), Balkwill et al. (2004), and Fritz et al. (2009), which reported that people can recognize emotions from unfamiliar cultures. Bowling (2013) suggested that similarities exist between musical and vocal expressions. Minor mode and sad speech are composed of smaller intervals, whereas major mode and joyful speech are composed of larger intervals. The acoustic cues used to express emotion are similar in speech and music, and emotional prosody can be cross-culturally recognized; thus, people in other cultures show emotional reactions to Western music that are similar to the emotional reactions shown by Western listeners (Fritz et al., 2009).

Furthermore, the major-mode music induced greater arousal than the minor-mode music. However, minor-mode music has been shown to induce more arousal than major-mode music in Western participants (van der Zwaag et al., 2011). Egermann et al. (2015) reported that arousal ratings were similar between Canadians and Pygmies who were naïve to Western music. The discrepant findings in this study may be due to the different stimuli used. The music materials used in the present studies were modified versions of “Bayer Piano Basic Courses.” The arousal-relevant factors, such as the style of the player, tempo, rhythm, and timbre, were controlled, and the only two independent variables were the musical modes and harmonic complexity. However, van der Zwaag et al. (2011) and Egermann et al. (2015) used musical pieces from popular songs and film soundtracks, and the tempo and rhythm were not standardized. Thus, the differences in the music materials may be responsible for the inconsistent results. In addition, the results of Experiment 2 showed that mode did not impact the dominance rating. In this study, the effects of mode on pleasure and arousal indicated that Western music modes had universal effects on emotion. Finally, the minor-mode music induced more tension than the major-mode music, which may be due to the structural characteristics of the minor mode. The root of the minor triad is more ambiguous and uncertain than the root of the major triad (Parncutt, 2014), which may result in feelings of disharmony and surprise.

### The Effects of Harmonic Complexity on Psychological Perception

Although the music with slightly higher harmonic variance and complexity produced higher liking ratings than the music with slightly lower harmonic variance in Experiment 1, the number of stimuli and the size of the sample were relatively small. Thus, the results of Experiment 2, namely, that the harmonic complexity did not influence perceptions, were more reliable. Bigand et al. (1996) demonstrated that more complex chord sequences elicit more tension, which is inconsistent with our findings. There are two possible explanations for this inconsistency. First, Bigand et al. (1996) used single chord sequences, whereas the present studies embedded the chord sequences into long excerpts. The influence of harmonic complexity on tension may be weakened by the context. Second, since the effects on pleasure and liking ratings were also driven by the number of hours of Western music listening, differences in familiarity with Western music may lead to differences in the perception of harmonic complexity between Chinese and Western listeners.

### The Role of Prior Knowledge of Western Music in Psychological Perception

Previous research on Westerners revealed that listeners without any formal music training can acquire some musical capacities by everyday exposure to music through an implicit learning process (Tillmann et al., 2000; Bigand and Poulin-Charronnat, 2006). As a result, listeners were able to implicitly encode the structures, tonality and stabilities and functions of Western music (Tillmann et al., 2008; Brattico et al., 2010). The participants in the current studies had different degrees of exposure to Western music (number of hours of Western music listening). We took participants’ prior knowledge of Western music as a covariate to test whether it had a key role in musical mode perception. Liking ratings (Experiment 1) and pleasure (Experiment 2) induced by musical modes were influenced by prior knowledge of Western music. Therefore, Chinese participants’ preference for musical mode was influenced by previous exposure to Western music, consistent with their Western counterparts. Interestingly, arousal, dominance, and tension were not affected by prior knowledge of Western music. Future human perception research on the cross-cultural characteristics of musical mode should consider the effect of prior knowledge.

### Limitations

The present investigation is limited in two respects. First, some of the participants had some exposure to Western music; thus, they were not completely naïve to Western music. The results showed that Western music expertise influenced liking ratings (Experiment 1) and pleasure (Experiment 2). A controlled cross-cultural study involving participants who are completely unfamiliar with music from other cultures should be performed (Egermann et al., 2015). Second, the samples in the present studies were slightly small for behavioral studies.

## Conclusion

The current study supports the hypothesis that musical mode has a cross-cultural influence on the psychological perception of Western music among Chinese people. The major-mode music induced greater pleasure, arousal, and liking ratings than the minor-mode music, whereas the minor-mode music induced greater tension than the major-mode music. Harmonic complexity did not affect music perception.

## Author Contributions

JS and LF designed the experiment. JS and LF prepared materials and performed the experiment. JS and LF analyzed the data. LF, JS, and NC wrote the paper.

## Conflict of Interest Statement

The authors declare that the research was conducted in the absence of any commercial or financial relationships that could be construed as a potential conflict of interest.
